# Identification of two novel HIV-1 circulating recombinant forms of CRF111_01C and CRF116_0108 in southwestern Yunnan, China

**DOI:** 10.1080/21505594.2021.2010399

**Published:** 2021-12-24

**Authors:** Mei Ye, Xin Chen, Lin Duo, Jin Ma, Le Cao, Chiyu Zhang, Yong-Tang Zheng

**Affiliations:** a Key Laboratory of Animal Models and Human Disease Mechanisms of the Chinese Academy of Sciences, Kiz-cuhk Joint Laboratory of Bioresources and Molecular Research in Common Diseases, Center for Biosafety Mega-Science, Kunming Institute of Zoology, Chinese Academy of Sciences, Kunming, Yunnan, China; b Kunming College of Life Science, University of Chinese Academy of Sciences, Kunming, Yunnan, China; c Department of Pathogenic Biology, School of Basic Medical Sciences, Gannan Medical University, Ganzhou, Jiangxi, China; d Yunnan Fuwai Cardiovascular Hospital, Kunming, Yunnan, China; e Cangyuan Va Autonomous County People’s Hospital, Lincang, Yunnan, China; f Shanghai Public Health Clinical Center, Fudan University, Shanghai, China

**Keywords:** Human immunodeficiency virus type 1, heterosexual contacts, circulating recombinant forms, China, Yunnan

## Abstract

Yunnan, the region hardest hit by HIV/AIDS in China, is also an area with the most abundant HIV-1 genetic diversity. A large number of novel HIV-1 circulating recombinant forms (CRFs) and unique recombinants were identified among injection drug users in Yunnan; however, few were found among sexual contacts. Here, we obtained 15 near full-length genome sequences (NFLGs) from HIV-1 seropositive heterosexual contacts in Yunnan who received antiretroviral therapy during the period from 2014 to 2016. Phylogenetic analysis showed that six NFLGs belonged to CRF01_AE (n = 3) and CRF106_cpx (n = 3), and the other nine sequences were novel inter-subtype recombinants. Of the recombinants, two novel CRFs (CRF111_01 C (n = 4) and CRF116_0108 (n = 4)) and one CRF106_cpx variant (n = 1) were identified. CRF111_01 C had a CRF01_AE backbone with seven subtype C fragments inserted into the *gag, pol, vif, env, nef* and *3ʹLTR* regions. CRF116_0108 had a CRF08_BC backbone with a CRF01_AE fragment inserted into the *pol, tat, rev, vif, vpr, vpu* and *env* regions. Phylogeographic analyses estimated that CRF111_01 C and CRF116_0108 originated approximately 1995.7–1998.6 and 1991.7–1993.7, respectively. These identifications of two novel HIV-1 CRFs highlighted the importance of continuous surveillance in heterosexual contacts and other high-risk groups in this region and the surrounding regions.

## Introduction

The human immunodeficiency virus (HIV) pandemic continues to be a major global public health issue. At the end of 2019, approximately 38.0 million people globally were living with HIV/AIDS, 1.7 million people became newly infected with HIV-1 and around 690,000 people died of AIDS-related illnesses worldwide, according to the Joint United Nations Programme on HIV/AIDS. In China, heterosexual transmission is the most predominant route for HIV-1 infection [1]. In 2019, 73.7% of new HIV infections were estimated to be caused by heterosexual transmission. Furthermore, HIV-1 is easily transmitted from high-risk groups (e.g., injection drug users (IDUs) and female sex workers) to their husband/wife and/or sexual partners [2].

**Figure 1.**
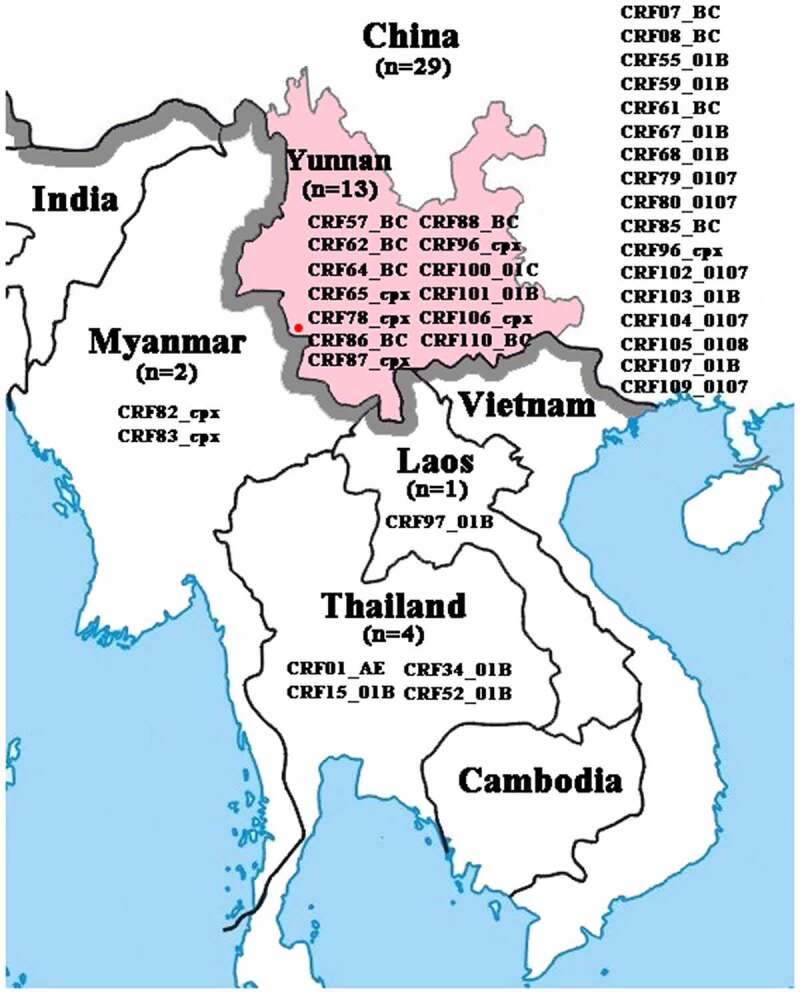
Geographical location of Yunnan and CRFs identified in Southeast Asian countries. The red dot indicates the sampling site

HIV is divided into type 1 (HIV-1) and type 2 (HIV-2), and HIV-1 is further divided into four groups (M, O, N and P) [3]. The group M is the world’s major epidemic pathogen of HIV/AIDS, and was further classified into ten subtypes (A, B, C, D, F, G, H, J, K and L) and a series of circulating recombinant forms (CRFs) and unique recombinant forms (URFs) [4]. Despite many advances in the diagnosis and antiretroviral treatment of HIV-1, the high diversity and recombination complexity of HIV-1 have long been major concern issues, challenging the HIV-1 surveillance, diagnosis, antiretroviral therapy, and vaccine development [5,6]. HIV-1 inter-subtype recombination has become more diversified over time [7]. As a result, 110 distinct CRFs and a large number of URFs have been documented globally due to the co-circulation of multiple HIV-1 subtypes so far [7,8]. There are currently 47 CRFs formed by recombination among subtypes B, C and CRF01_AE, 35 of which were first identified in China and neighboring Southeast Asian countries (Figure 1), such as Myanmar [9], Laos [10] and Thailand [11–13]. We previously reported an extremely high proportion of HIV-1 recombinants among IDUs in the China-Myanmar border region and identified four new CRFs (CRF82_cpx, CRF83_cpx, CRF87_cpx and CRF88_BC) [9,14–16]. In addition, a complicated HIV-1 CRF01_AE/B/C recombinant isolated from a long-distance truck driver in northern Myanmar was identified [17]. More than ten HIV-1 subtypes/CRFs and a series of inter-subtype recombinants were found among the newly diagnosed HIV-1 infected individuals in Yunnan, and the recombinants accounted for 86.0% of total infections [18]. These studies indicated the ongoing generation of complex HIV-1 recombinants among subtypes B, C and CRF01_AE in northern Myanmar as well as in Yunnan [14,15,18,19].

The HIV-1 epidemic in China has been driven by CRF01_AE, CRF07_BC and CRF08_BC in recent years, which caused the co-circulation of these CRFs with previously dominant subtypes (e.g., B and C). As a consequence, some second-generation recombination forms (2^nd^-CRFs) are generated from these CRFs and subtypes. In particular, an increasing number of the 2^nd^-CRFs by CRFs were identified, including CRF79_0107 [20], CRF80_0107 [21], CRF102_0107 [22], CRF104_0107 [23], CRF105_0108 [24], and CRF109_0107 [25]. A recent systematic review and meta-analysis that focuses on the heterosexual contacts in China showed a significantly higher prevalence of CRF01_AE and CRF08_BC in heterosexual groups in the southern provinces and southwest provinces, respectively [1]. In particular, HIV-1 CRF01_AE and CRF08_BC were two main circulating HIV-1 strains in IDUs and the heterosexual contacts in Yunnan for years [18,26]. However, only one 2^nd^-CRF105_0108 was identified among sexual contacts (four homosexual contacts and one heterosexual contact) in Sichuan [24]. Furthermore, the first report of a novel CRF100_01 C consisting of CRF01_AE and C was identified among heterosexual contacts [27]. Whether there are new CRFs and new 2^nd^-CRFs to be formed by these CRFs and subtypes among the heterosexuals remains less investigated. In this study, we reported two newly identified CRFs, CRF111_01 C and CRF116_0108, among the heterosexual group in Yunnan, China.

## Materials and methods

### Study samples

A survey was conducted among HIV-1 infected individuals who received antiretroviral therapy at Cangyuan Wa Autonomous County People’s Hospital between 2014 and 2016 (Figure 1). The original study protocol was reviewed and approved by the Ethics Committee of Kunming Institute of Zoology, Chinese Academy of Sciences (approval number: SWYX-2013023; date: 6 September 2013). All participants provided written informed consent.

### DNA extraction and PCR of provirus DNA

Genomic DNA was extracted from 200 μl of blood cell samples using the Blood Genomic DNA Mini Kit (CW Bioteck Co., Ltd., Beijing, CW2087M). The near full-length genome (NFLG) of HIV-1 was amplified by nested-PCR employing a TransTaq DNA Polymerase High Fidelity kit (Beijing TransGen Biotech Co., Ltd., AP131-13) into three overlapping fragments (A1 (187–3556) or A2 (769–3338), B (3282–6294), and C (5843–9558) relative to the HXB2 genome) using primers as described previously [10,28]. The primers for PCR are listed in Table S1. The amplified PCR products were analyzed by 1% agarose gel electrophoresis, and the PCR-positive products were then purified and sequenced by Tsing Ke Biotech (Kunming, China) Co., Ltd.

### Phylogenetic and recombination analyses

Three obtained fragments were assembled using SeqMan in the DNAstar software package. Obtained NFLG sequences were aligned by the HIVAlign (https://www.hiv.lanl.gov/content/sequence/VIRALIGN/viralign.html) with HIV-1 reference sequences of different subtypes and CRFs (all available reference sequences of HIV-1 group M, CRF01_AE, CRF07_BC, CRF08_BC, CRF100_01 C, CRF105_0108 and CRF106_cpx).

Phylogenetic tree analysis was conducted by MEGA 7.0 using the maximum-likelihood (ML) method under the general time reversible model with a gamma-distributed model of among site rate variation using four rate categories (GTR+I + G) nucleotide substitution model with 1,000 bootstrap replications; the best nucleotide substitution model was selected by using the Akaike information criterion implemented in jModelTest 2.1.7. Furthermore, all the study sequences were further analyzed for the presence of recombination, and possible recombination breakpoints were identified by using the bootscanning approach as implemented in SimPlot v3.5.1 software, as described previously [9]. To confirm the recombination breakpoints of novel HIV-1 CRFs, ML trees were reconstructed with each sub-region using the same phylogenetic analysis. The genomic maps of the HIV-1 recombinant forms were visualized with the Recombinant HIV-1 Drawing Tool.

### Next-generation sequencing

To explore whether secondary recombination occurs in 14YN252, we targeted amplification of the recombination fragment (HXB2:6024–7381) from 14YN251 and 14YN252. In brief, the first round of PCR products of 14YN251 and 14YN252 fragment C were used as templates, and then the second round of PCR was performed using primers N1F (5ʹ-ATCARAHTCYTRTAYCAAAGCAGTAAGTA-3ʹ) and ED33 (5ʹ-TTACAGTAGAAAAATTCCCCTC-3ʹ) to amplify the 1357 bp sequence, including the HIV-1 partial *tat-rev-vpu-env* gene. PCR products were then transported to Seqhealth Technology Co., Ltd. (Wuhan, China) for DNA quality testing, library preparation and next-generation sequencing (NGS). Library preparation was performed with a VAHTS Universal DNA Library Prep Kit for Illumina V3 (Cat. # ND607, Vazyme Biotech Co., Ltd, Nanjing, CN). DNA libraries were then paired-end sequenced (2 × 150) with a sequencing depth of 1 G using the Illumina HiSeq X Ten platform (Illumina Inc., San Diego, CA, USA). The raw sequence reads were filtered for low-quality reads using Kneaddata with default settings, and the parameter “–trimmomatic-option” was ‘SLIDINGWINDOW: 4:20 MINLEN: 100ʹ [29]. The clean reads were subsequently de-novo assembled using Megahit [30]. To calculate the coverage, the clean reads were aligned to assembled contigs using bowtie2 to obtain the information of reads mapping; then, Checkm was used to calculate the number of mapped reads and coverage [31,32]. Finally, all assembled contigs were aligned to reference genomes using Bowtie2 to select object-related contigs.

### Molecular clock signal and Bayesian phylogenetic analyses

To explore the origin time and phylogenetic relationships of the novel HIV-1 CRFs, sequence datasets including two and three different genomic regions with different subtype-origin were selected from CRF111_01 C and CRF116_0108, respectively, and subjected to Bayesian phylogenetic analyses using BEAST v.1.10.4. Available subtype C, CRF01_AE and CRF08_BC sequences with known sampling years from China, Myanmar, Thailand, Vietnam, India, Central African Republic, South Africa, Brazil and Ethiopia were retrieved from the Los Alamos HIV Database (http://www.hiv.lanl.gov) and also included in the Bayesian phylogenetic analyses. The best substitution model (GTR+I + G) for each sequence dataset was inferred using Jmodeltest v2.1.7. The temporal signal of each dataset was evaluated by calculating the correlation coefficient between the root-to-tip divergence and sampling date with TempEst v1.5.1 [33]. The coefficient of correlation between root-to-tip divergence versus sampling date (Table S2) supports the presence of a temporal signal and the use of a molecular clock analysis in BEAST. We tested different molecular clock model (strict and uncorrelated lognormal relaxed molecular clock) and tree prior (constant size, exponential growth and Bayesian skyline coalescent) combinations using the marginal likelihoods calculated by path sampling/stepping-stone sampling [34]. All analyses were run for 100 million generations, with sampling every 10,000 generations. Then, we sampled 20 path steps with a chain length of 5 million, with power posteriors determined according to evenly spaced quantiles of a Beta (0.3, 1.0) distribution. An uncorrelated lognormal relaxed clock model with Bayesian skyline or exponential growth tree prior was identified as the best combination of models (Table S3). The maximum clade credibility (MCC) trees were inferred under the optimal BEAST model combination and best fit nucleotide substitution model using a Bayesian Markov Chain Monte Carlo (MCMC) approach. To ensure an effective sample size (ESS) > 200, MCMC analysis for each dataset was run for 300 million generations and sampling every 30,000 generations. The MCC trees were generated using TreeAnnotator in the BEAST v1.10.4 package, which were further edited and visualized with the program Figtree v1.4.3.

## Data availability

All HIV-1 sequences generated in this study were deposited in the GenBank database (accession numbers MT624743-MT624757) and NCBI SRA database (accession number: PRJNA723921).

## Results

### Social-demographic characteristics of the study samples

We obtained 15 NFLGs from HIV-1 positive samples collected from heterosexual contacts during 2014–2016. The social-demographic information of 15 individuals with available NFLGs, including sample years, gender, age, ethnic group, education level, and marriage status, is summarized in Table 1.

**Table 1.** Demographic characteristics of 15 HIV-1 infected individuals

Sequence name	Sampling year	Sex	Age	Ethnic group	Education	Marriage	Accession number
14YN68	2014	male	34	Wa	illiteracy	unmarried	MT624743
14YN251	2014	female	24	Wa	Primary school	married	MT624744
14YN252	2014	male	35	Wa	Primary school	married	MT624745
14YN526	2014	female	24	Dai	illiteracy	unmarried	MT624746
14YN263	2014	male	33	Wa	Junior high school	married	MT624747
14YN264	2014	female	28	Wa	Junior high school	married	MT624748
16YN253	2016	male	46	Han	illiteracy	married	MT624749
16YN424	2016	female	42	Wa	Junior high school	married	MT624750
16YN29	2016	female	39	Wa	illiteracy	married	MT624751
16YN604	2016	female	40	Wa	illiteracy	divorced	MT624752
16YN642	2016	female	39	Wa	illiteracy	divorced	MT624753
16YN652	2016	male	36	Han	Junior high school	married	MT624754
16YN732	2016	female	32	Yi	Junior high school	married	MT624755
16YN764	2016	female	59	Wa	illiteracy	married	MT624756
16YN666	2016	female	27	Wa	Junior high school	unmarried	MT624757

### Identification of the HIV-1 CRF111_01 C and CRF116_0108

**Figure 2.**
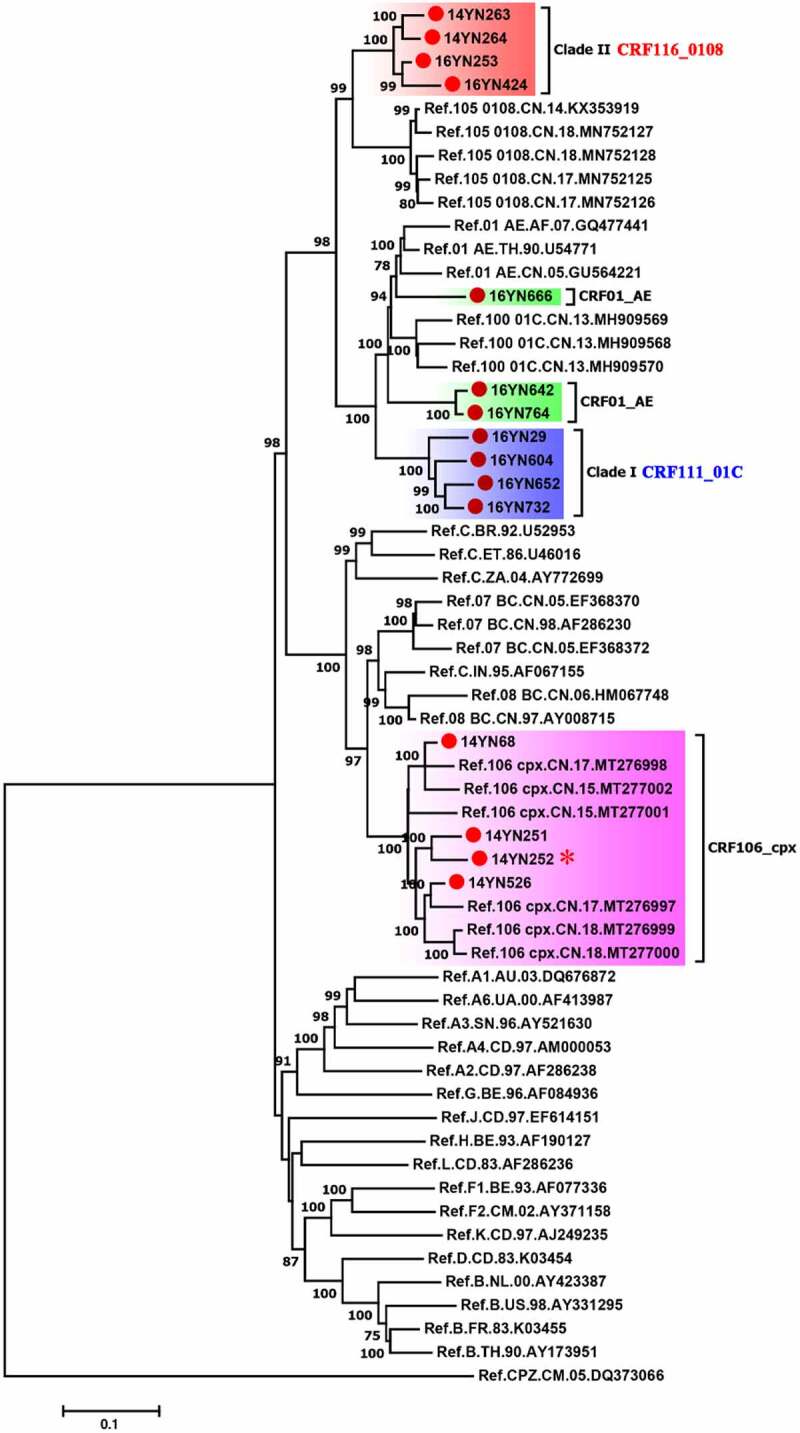
The maximum likelihood tree of 15 HIV-1 near full-length genomic sequences from Yunnan, China. All 15 sequences obtained in this study are highlighted with red circles. The blue and red shadows indicate the clades of CRF111_01 C and CRF116_0108, respectively. The red star indicates that 14YN252 belongs to the CRF106_cpx variant

A near full-length genome phylogenetic tree divided 8 of 15 NFLGs into two clades I (16YN29, 16YN604, 16YN652 and 16YN732) and II (14YN263, 14YN264, 16YN253 and 16YN424). Phylogenetic analysis showed that the two clades did not cluster with any clusters formed by known HIV-1 subtypes/CRFs, implying that the 8 sequences were likely to be new subtypes/CRFs (Figure 2). In the phylogenetic tree, clade I was located outside the cluster of CRF01_AE (Figure 2). Bootscan analyses indicated that the sequences in clade II included seven CRF01_AE fragments and seven C fragments separated by 13 recombination breakpoints (Figure S1A). Because these sequences had the same recombination pattern, they were defined as a new CRF111_01 C (Figure S1A). The genomic map of CRF111_01 C was as follows: I CRF01_AE (243–1448 nt), II C (1449–1814 nt), III CRF01_AE (1815–3090 nt), IV C (3091–3388 nt), V CRF01_AE (3389–4656 nt), VI C (4657–4907 nt), VII CRF01_AE (4908–5189 nt), VIII C (5190–5317 nt), IX CRF01_AE (5318–7001 nt), X C (7002–7584 nt), XI CRF01_AE (7585–9037 nt), XII C (9038–9145 nt), XIII CRF01_AE (9146–9387 nt), and XIV C (9388–9521 nt) (Figure S1B). Sub-region ML trees based on each segment of CRF111_01 C further confirmed the results of recombination analysis (Figure S2).

Four sequences in clade II were near the reference sequences of CRF105_0108 (Figure 2). Bootscan analyses showed that these sequences were composed of CRF01_AE and CRF08_BC, with two recombination breakpoints (Figure S3A). Because these sequences shared the same recombination pattern, they were defined as a new CRF116_0108 (Figure S3A). The genomic structure of the CRF116_0108 was as follows: I CRF08_BC (826–4683 nt), II CRF01_AE (4684–8727 nt), and III CRF08_BC (8728–9521 nt) (Figure S3B). Sub-region ML trees based on each segment of CRF116_0108 confirmed the results of recombination analysis (Figure S4).

Moreover, four sequences (14YN68, 14YN251, 14YN252 and 14YN526) tightly clustered with the reference sequences of CRF106_cpx, supported by a bootstrap value of 100% (Figure 2), indicating that they belong to CRF106_cpx. Phylogenetic and bootscan analyses revealed that the other 3 strains (16YN642, 16YN666 and 16YN764) belonged to CRF01_AE (Figure 2, Figure S5A).

### The identification of a CRF106_cpx variant

**Figure 3.**
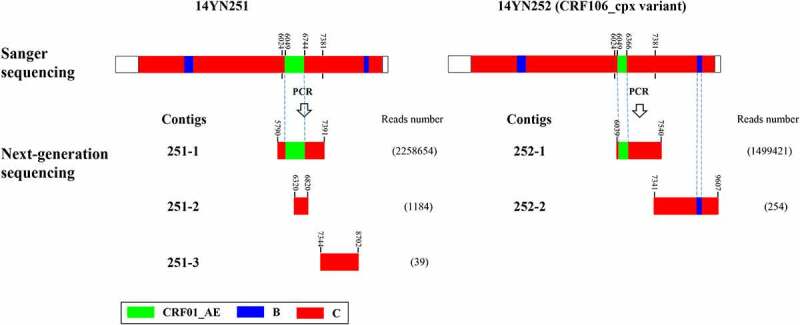
Genomic structural maps for quasispecies of HIV-1 (HXB2:6024–7381) from 14YN251 and 14YN252

14YN251 and 14YN252 were regular sexual partners, and two NFLGs from them closely clustered together, suggesting direct transmission of the virus between them. Because 14YN251 self-reported being infected earlier than 14YN252, the virus was more likely transmitted from 14YN251 to 14YN252. Interestingly, however, we found that the recombination pattern of 14YN252 was slightly different from that of 14YN251 and other CRF106_cpx strains (Figure S5B), suggesting the occurrence of further virus recombination in 14YN252. To trace the occurrence of secondary recombination in 14YN252, we conducted next-generation sequencing of a specific recombination fragment (HXB2:6024–7381) that carries distinct recombination breakpoints and distinguishes 14YN251 and 14YN252 (Figure 3). Three and two contig sequences were obtained from 14YN251 and 14YN252, respectively (Figure 3). In 14YN251, two different contig sequences that correspond to the targeted fragment were obtained, indicating the co-circulation of at least two HIV-1 variants. The most predominant contig sequence had a very high read abundance (2,258,654) and shared a consistent CRF106_cpx recombination pattern with the sequence obtained by Sanger sequencing. Another contig sequence with very low read abundance represents a minor variant and belongs to subtype C. In 14YN252, only one contig sequence corresponded to the targeted fragment and shared the same recombination pattern with the sequence (CRF106_cpx-related recombinant, or CRF106_cpx variant) obtained by Sanger sequencing, indicating that the secondary generation recombination occurred earlier before sampling, and the CRF106_cpx-related recombinant completely replaced CRF106_cpx as the dominant strain. The two other contig sequences were outside the targeted region, and might be derived from HIV-1 proviral DNA.

### Evolutionary history of HIV-1 CRF111_01 C and CRF116_0108

**Figure 4.**
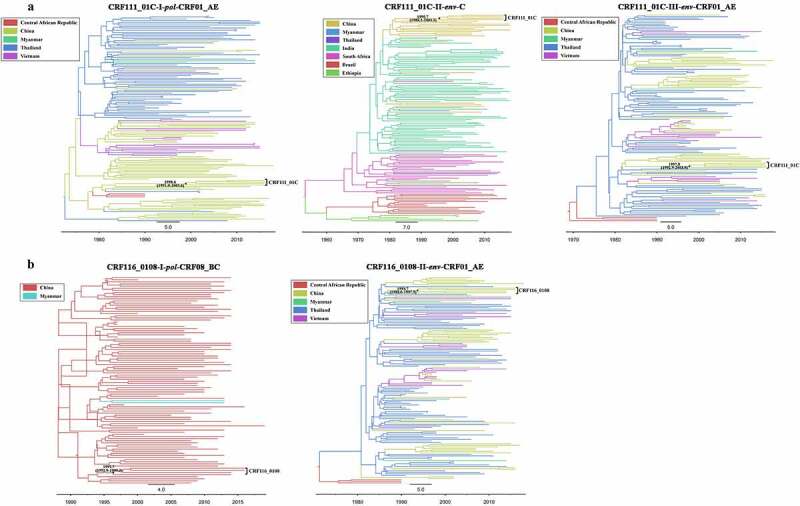
Maximum clade credibility (MCC) trees of the HIV-1 CRF111_01 C and CRF116_0108. (a) The reconstructed MCC tree based on three genomic segments of CRF111_01 C, including the *pol* region of subtype CRF01_AE origin (3600–4600 nt in HXB2), *env* region of subtype C origin (7002–7584 nt in HXB2) and *env* region of subtype CRF01_AE origin (7790–8790 nt in HXB2). (b) The reconstructed MCC tree based on two genomic segments of CRF116_0108, including the *pol* region of subtype CRF08_BC origin (3200–4200 nt in HXB2) and the *env* region of subtype CRF01_AE origin (7002–8002 nt in HXB2). Different tree branch colors represent different countries. The black solid dots on the trees indicate the tMRCA of CRF111_01 C and CRF116_0108

The Bayesian analyses based on three segments including the *pol* region of CRF01_AE origin (HXB2: 3600–4600 nt), *env* region of subtype C origin (HXB2: 7002–7584 nt) and *env* region of CRF01_AE origin (HXB2: 7790–8790 nt) showed that the most recent common ancestor (tMRCA) of CRF111_01 C were 1998.6 (95% confidence interval (CI): 1991.0–2005.6), 1995.7 (95% CI: 1988.2–2003.1) and 1997.8 (95% CI: 1992.9–2003.0), respectively (Figure 4a), indicating that CRF111_01 C originated during 1995.7–1998.6. tMRCA of CRF116_0108 was estimated as 1991.7 (95% CI: 1992.9–1999.3) and 1993.7 (95% CI: 1988.0–1997.9) based on CRF08_BC (HXB2: 3200–4200 nt) and CRF01_AE (HXB2: 7002–8002 nt) segments, respectively (Figure 4b). Therefore, CRF116_0108 was inferred to originate in approximately 1991.7–1993.7.

## Discussion

Multiplex HIV-1 subtypes/CRFs are co-circulating in the China-Myanmar border area [14,15,19,35,36]. Accompanied by the decrease in the prevalence of pure HIV-1 group M subtypes (such as B and C), the prevalence of various recombinant forms is progressively increasing. In recent years, a large number of HIV-1 inter-subtype recombinants were identified among IDUs in this area, and the vast majority of them were complex recombinants formed by subtypes of B, C and CRF01_AE, including some newly identified CRFs, CRF82_cpx, CRF83_cpx and CRF87_cpx [9,15,16]. However, few CRFs were identified among the heterosexuals, the major high-risk group for HIV-1 infection in Yunnan and having a high prevalence of both CRF01_AE and CRF08_BC. This study identified two novel CRFs, named CRF111_01 C and CRF116_0108 from the heterosexuals in Yunnan. Although the biological, transmission and pathogenic features remain to be evaluated, the identification of the two novel CRFs has important implications for HIV-1 evolution, transmission, diagnosis and treatment [5,6].

The proportion of heterosexual transmission of HIV-1 has rapidly increased in recent years. However, HIV-1 transmission between regular sexual partners is often ignored [37]. In 2011, the proportion of heterosexual transmission of HIV-1 in China was estimated to be 46.5%, of which about a quarter was sexual transmission between regular sexual partners [38]. Occasional sex with other sexual partners and other high-risk factors for HIV-1 transmission increase the risk of HIV-1 transmission within a family. A survey conducted among blood transfusion recipients in Hebei showed that the rate of intra-familial transmission of HIV-1 was up to 30.7% (81/264), of which regular sexual partners’ transmission and mother-to-child transmission reached 20.9% (53/254) and 28.1% (46/164), respectively [39]. Currently, although some interventions have been employed to decrease HIV-1 transmission among the pre-marital population and to avoid mother-to-child transmission, the measures to reduce HIV-1 intra-familial transmission among high-risk regular sexual partners are very limited, especially in some areas of Yunnan Province, China, which led to an increased prevalence of HIV-1 among this group.

Lincang Prefecture, bordering the northeastern regions of Myanmar, is a “hot spot” region for HIV-1 recombination [27,40]. A recent study reported that the vast majority (93.8%) of HIV-1 positive individuals initiating antiretroviral therapy in Lincang Prefecture were the heterosexuals. CRF08_BC was the most predominant HIV-1 genotype (70.2%, 226/322) among them, followed by some URFs (10.6%, 34/322) [40]. In this study, we first identified two new HIV-1 CRFs (CRF111_01 C and CRF116_0108) from the heterosexuals in Cangyuan County of Lincang Prefecture according to 15 NFLGs and detected co-circulation of the two new CRFs (26.7% both) with CRF106_cpx (26.7%, including a CRF106_cpx variant) and CRF01_AE (20.0%). In spite that no CRF08_BC and other URFs were found in this cohort, our results clearly supported an on-going increase of HIV-1 genetic diversity among the heterosexuals in this region. On the other hand, the NGS data suggested that at least the individual 14YN251 was co-infected by CRF106_cpx and subtype C, implying the presence of sexual activity outside marriage with occasional sexual partners. Given the high potential of HIV-1 recombination, co-circulation of four CRFs among the heterosexuals with regular and occasional sexual partners will inevitably result in the generation of new 2^nd^-CRFs and even 3^rd^-CRFs formed by these CRFs.

Yunnan is a hotspot for HIV-1 inter-subtype recombination, mainly due to the co-circulation of HIV-1 subtypes B, C and CRF01_AE among IDUs [19]. A large number of URFs were detected among IDUs in Yunnan, and some of them were later identified as CRFs, including CRF57_BC, CRF87_cpx, CRF88_BC, CRF96_cpx, CRF101_01B and CRF110_BC. The molecular epidemiology of HIV-1 recombinants in Yunnan was characterized by three main features. First, the vast majority of URFs and CRFs were generated by the recombination between B and C [19], as well as among B, C and CRF01_AE [16,35,36], but very few URFs and CRFs were derived from the recombination between subtypes C and CRF01_AE. Second, the vast majority of URFs and CRFs originated and were mainly circulating among IDUs, but few from the heterosexuals. Third, accompanied by the co-circulation of CRF01_AE, CRF07_BC and CRF08_BC, six 2^nd^-CRFs (CRF79_0107, CRF80_0107, CRF102_0107, CRF104_0107, CRF105_0108, and CRF109_0107) were generated [20–25]. The vast majority of the 2^nd^-CRFs were derived from CRF01_AE and CRF07_BC, but only one (CRF105_0108) that was first identified in Sichuan (outside Yunnan) was formed by CRF01_AE and CRF08_BC [24]. In this study, the identification of one new CRF (CRF111_01 C: 26.7%) derived from CRF01_AE and C, and one new 2^nd^-CRF (CRF116_0108: 26.7%) derived from CRF01_AE and CRF08_BC, as well as the finding of the newly identified CRF106_cpx (26.7%) among 15 heterosexuals, might imply a changing trend of HIV-1 molecular epidemiology among the heterosexuals, at least in Lincang Prefecture of Yunnan, which has important epidemiological implications.

Among CRFs identified in China, only three (CRF07_BC, CRF08_BC and CRF55_01B) caused the epidemic since their origin, while the others were just associated with sporadic infections. CRF07_BC and CRF08_BC were the earliest identified CRFs in China, both of which originated among IDUs in Yunnan in early 1990s and later spread to heterosexuals and homosexuals and other regions of China [41]. CRF07_BC has a nationwide prevalence. It remained the most predominant HIV-1 strain among IDUs and was becoming the most predominant HIV-1 among men who have sex with men (MSM) [42], while the prevalence of CRF08_BC was mainly restricted to some regions (e.g. Yunnan and Guangxi) [18,40,43]. CRF55_01B originated among MSM in about 2001 [44] and showed an increasing prevalence trend in recent years. CRF111_01 C and CRF116_0108 were estimated to originate in about 1995.7–1998.6 and 1991.7–1993.7, respectively, implying a long history. The origin time of CRF111_01 C and CRF116_0108 was slightly later than those of CRF07_BC and CRF08_BC, but obviously earlier than that of CRF55_01B, and other CRFs (e.g., CRF62_BC) [41,44,45]. The main reason for why CRF111_01 C and CRF116_0108 were not found for a long time might be that their prevalence was mainly restricted to the heterosexuals in Cangyuan County, and this cohort was previously ignored. However, as evidenced by the prevalence and spread of CRF07_BC and CRF01_AE, HIV-1 was easily transmitted among different high-risk groups (e.g., IDUs, the heterosexuals and MSM) and in different regions. Therefore, CRF111_01 C and CRF116_0108 were not excluded to circulate in other high-risk groups (e.g., IDUs and/or MSM) and the surrounding regions. A large-scale molecular epidemiological investigation should be performed to see whether there are more infections with CRF111_01 C and CRF116_0108 among the heterosexuals and other high-risk groups in Cangyuan and the surrounding regions. Furthermore, a long-time monitoring should also be encouraged to track the transmission of the two new CRFs, and to further evaluate their transmission risk from the heterosexuals to other high-risk groups and from Cangyuan to other regions.

There are two limitations in our study. The first one is the relatively small sample size. Despite the small sample size, we identified two new HIV-1 CRFs (CRF111_01 C and CRF116_0108) according to the criterion of a new HIV-1 CRF and found the co-circulation of four CRFs (CRF01_AE, CRF106_cpx, CRF111_01 C and CRF116_0108) in the heterosexuals in Cangyuan County of Lincang Prefecture. The second is that the samples were collected during 2014–2016, which might not reflect the current prevalence pattern of HIV-1 among the heterosexuals. As mentioned above, whether the two new CRFs have caused a wide epidemic in the heterosexuals and even other high-risk groups (e.g. IDUs) in this region and/or surrounding regions needs to be concerned.

In summary, we identified two novel HIV-1 CRFs (CRF111_01 C and CRF116_0108) from the heterosexual contacts in Cangyuan County of Yunnan Province, and estimated that the origin times of CRF111_01 C and CRF116_0108 were in around 1995.7–1998.6 and 1991.7–1993.7, respectively. Furthermore, we detected the co-circulation of four CRFs in this cohort. Given the on-going increase of HIV-1 genetic diversity in Yunnan province and the increasing prevalence trend of some newly identified CRFs (e.g. CRF07_BC, CRF08_BC and CRF55_01B) in China, the identification of two novel CRFs has important epidemiological implications, which highlights the importance of continuous surveillance.

## Supplementary Material

Supplemental Material

## Data Availability

The datasets presented in this study can be found in online repositories. These data can be found here: https://www.ncbi.nlm.nih.gov/nuccore/MT624743.

## References

[1] Xiao P, Li J, Fu G, et al. Geographic distribution and temporal trends of HIV-1 subtypes through heterosexual transmission in China: a systematic review and meta-analysis. Int J Environ Res Public Health. 2017;14:830. 28737729 10.3390/ijerph14070830 PMC5551268

[2] Zheng S. The growing threat of China’s HIV epidemic. Lancet Public Health. 2018;3:e311. 29976325 10.1016/S2468-2667(18)30098-7

[3] Robertson DL, Anderson JP, Bradac JA, et al. HIV-1 nomenclature proposal. Science. 2000;288:55–56. 10766634 10.1126/science.288.5463.55d

[4] Yamaguchi J, Vallari A, McArthur C, et al. Brief report: complete genome sequence of CG-0018a-01 establishes HIV-1 subtype L. J Acquir Immune Defic Syndr. 2020;83:319–322. 31693506 10.1097/QAI.0000000000002246 PMC7012332

[5] Hemelaar J, Elangovan R, Yun J, et al. Global and regional epidemiology of HIV-1 recombinants in 1990-2015: a systematic review and global survey. Lancet HIV. 2020;7:e772–e781. 33128904 10.1016/S2352-3018(20)30252-6

[6] Li YX, Chen X, Zhao YJ, et al. A rapid variant-tolerant reverse transcription loop-mediated isothermal amplification assay for point of care detection of HIV-1. Analyst. 2021;146:5347–5356. 34323889 10.1039/d1an00598g

[7] Hemelaar J, Elangovan R, Yun J, et al. Global and regional molecular epidemiology of HIV-1, 1990-2015: a systematic review, global survey, and trend analysis. Lancet Infect Dis. 2019;19:143–155. 30509777 10.1016/S1473-3099(18)30647-9

[8] Li J, Gao Q, Zhang M, et al. A newly emerging HIV-1 circulating recombinant form (CRF110_BC) comprising subtype B and C among intravenous drug users in Yunnan, China. J Infect. 2021;82:e8–e10. 10.1016/j.jinf.2020.12.015 33352212

[9] Chen X, Ye M, Duo L, et al. First description of two new HIV-1 recombinant forms CRF82_cpx and CRF83_cpx among drug users in Northern Myanmar. Virulence. 2017;8:497–503. 27574950 10.1080/21505594.2016.1226722 PMC5538334

[10] Chen X, Ye M, Wang Y, et al. Laos is affected by HIV CRF01_AE and the newly identified CRF97_01B. Virol Sin. 2020;35:538–547. 32232730 10.1007/s12250-020-00215-4 PMC7736601

[11] Tovanabutra S, Watanaveeradej V, Viputtikul K, et al. A new circulating recombinant form, CRF15_01B, reinforces the linkage between IDU and heterosexual epidemics in Thailand. AIDS Res Hum Retroviruses. 2003;19:561–567. 12908933 10.1089/088922203322230923

[12] Tovanabutra S, Kijak GH, Beyrer C, et al. Identification of CRF34_01B, a second circulating recombinant form unrelated to and more complex than CRF15_01B, among injecting drug users in northern Thailand. AIDS Res Hum Retroviruses. 2007;23:829–833. 17604547 10.1089/aid.2006.0300

[13] Liu Y, Li L, Bao Z, et al. Identification of a novel HIV type 1 circulating recombinant form (CRF52_01B) in Southeast Asia. AIDS Res Hum Retroviruses. 2012;28:1357–1361. 22269007 10.1089/aid.2011.0376 PMC3448130

[14] Yang R, Xia X, Kusagawa S, et al. On-going generation of multiple forms of HIV-1 intersubtype recombinants in the Yunnan Province of China. AIDS. 2002;16:1401–1407. 12131217 10.1097/00002030-200207050-00012

[15] Pang W, Zhang C, Duo L, et al. Extensive and complex HIV-1 recombination between B’, C and CRF01_AE among IDUs in South-East Asia. AIDS. 2012;26:1121–1129. 22333750 10.1097/QAD.0b013e3283522c97

[16] Hu Y, Wan Z, Zhou YH, et al. Identification of two new HIV-1 circulating recombinant forms (CRF87_cpx and CRF88_BC) from reported unique recombinant forms in Asia. AIDS Res Hum Retroviruses. 2017;33:353–358. 27762598 10.1089/aid.2016.0252 PMC5372766

[17] Zhou YH, Chen X, Liang YB, et al. Near full-length identification of a novel HIV-1 CRF01_AE/B/C recombinant in Northern Myanmar. AIDS Res Hum Retroviruses. 2015;31:845–850. 25970165 10.1089/AID.2015.0021

[18] Chen M, Ma Y, Chen H, et al. Spatial clusters of HIV-1 genotypes in a recently infected population in Yunnan, China. BMC Infect Dis. 2019;19:669. 31357947 10.1186/s12879-019-4276-9 PMC6664787

[19] Han X, An M, Zhao B, et al. High prevalence of HIV-1 intersubtype B’/C recombinants among injecting drug users in Dehong, China. PLoS One. 2013;8:e65337. 23741489 10.1371/journal.pone.0065337 PMC3669332

[20] Li Y, Feng Y, Li F, et al. Genome sequence of a novel HIV-1 circulating recombinant form (CRF79_0107) Identified from Shanxi, China. AIDS Res Hum Retroviruses. 2017;33:1056–1060. 28557610 10.1089/aid.2017.0066

[21] Zhang Y, Pei Z, Li H, et al. Characterization of a novel HIV-1 circulating recombinant form (CRF80_0107) among men who have sex with men in China. AIDS Res Hum Retroviruses. 2019;35:419–423. 30259751 10.1089/AID.2018.0226

[22] Li X, Wu J, Zhang Y, et al. Characterization of a novel HIV-1 second-generation circulating recombinant form (CRF102_0107) among men who have sex with men in Anhui, China. J Infect. 2019;79:612–625. 10.1016/j.jinf.2019.09.022 31622633

[23] Chang W, Zhang M, Ren Q, et al. HIV-1 genetic diversity and recombinant forms among men who have sex with men at a sentinel surveillance site in Xi’an City, China. Infect Genet Evol. 2020;81:104257. 32087346 10.1016/j.meegid.2020.104257

[24] Dong A, Liu L, Xiao L, et al. First detection of a circulating recombinant form of HIV-1 CRF01_AE/08_BC (CRF105_0108) with drug-resistant mutations in Sichuan, China. AIDS Res Hum Retroviruses. 2020;36:625–630. 32370607 10.1089/AID.2020.0034

[25] Wang X, Zhao J, Li X, et al. Identification of a novel HIV-1 second-generation circulating recombinant form CRF109_0107 in China. J Infect. 2020;81:816–846. 10.1016/j.jinf.2020.09.007 32946916

[26] Chen M, Jia MH, Ma YL, et al. The changing HIV-1 genetic characteristics and transmitted drug resistance among recently infected population in Yunnan, China. Epidemiol Infect. 2018;146:775–781. 29534773 10.1017/S0950268818000389 PMC9134363

[27] Feng Y, Zhang C, Zhang M, et al. First report of a novel HIV-1 recombinant form (CRF100_01C) comprising CRF01_AE and C among heterosexuals in Yunnan, China. J Infect. 2018;77:561–571. 10.1016/j.jinf.2018.10.009 30389423

[28] Nadai Y, Eyzaguirre LM, Constantine NT, et al. Protocol for nearly full-length sequencing of HIV-1 RNA from plasma. PLoS One. 2008;3:e1420. 18183300 10.1371/journal.pone.0001420 PMC2170516

[29] McIver LJ, Abu-Ali G, Franzosa EA, et al. bioBakery: a meta’omic analysis environment. Bioinformatics. 2018;34:1235–1237. 29194469 10.1093/bioinformatics/btx754 PMC6030947

[30] Li D, Liu CM, Luo R, et al. MEGAHIT: an ultra-fast single-node solution for large and complex metagenomics assembly via succinct de Bruijn graph. Bioinformatics. 2015;31:1674–1676. 25609793 10.1093/bioinformatics/btv033

[31] Langmead B, Salzberg SL. Fast gapped-read alignment with Bowtie 2. Nat Methods. 2012;9:357–359. 22388286 10.1038/nmeth.1923 PMC3322381

[32] Parks DH, Imelfort M, Skennerton CT, et al. CheckM: assessing the quality of microbial genomes recovered from isolates, single cells, and metagenomes. Genome Res. 2015;25:1043–1055. 25977477 10.1101/gr.186072.114 PMC4484387

[33] Rambaut A, Lam TT, Max Carvalho L, et al. Exploring the temporal structure of heterochronous sequences using TempEst (formerly Path-O-Gen). Virus Evol. 2016;2:vew007. 27774300 10.1093/ve/vew007 PMC4989882

[34] Baele G, Lemey P, Bedford T, et al. Improving the accuracy of demographic and molecular clock model comparison while accommodating phylogenetic uncertainty. Mol Biol Evol. 2012;29:2157–2167. 22403239 10.1093/molbev/mss084 PMC3424409

[35] Feng Y, Wei H, Hsi J, et al. Identification of a novel HIV Type 1 circulating recombinant form (CRF65_cpx) composed of CRF01_AE and subtypes B and C in Western Yunnan, China. AIDS Res Hum Retroviruses. 2014;30:598–602. 24279591 10.1089/aid.2013.0233 PMC4046203

[36] Song Y, Feng Y, Miao Z, et al. Near-full-length genome sequences of a novel HIV-1 circulating recombinant form, CRF01_AE/B’/C (CRF78_cpx), in Yunnan, China. AIDS Res Hum Retroviruses. 2016;32:601–606. 26885715 10.1089/AID.2015.0351

[37] Eshleman SH, Hudelson SE, Redd AD, et al. Analysis of genetic linkage of HIV from couples enrolled in the HIV prevention trials network 052 trial. J Infect Dis. 2011;204:1918–1926. 21990420 10.1093/infdis/jir651 PMC3209811

[38] Chinese Ministry of Health, UNAIDS, WHO. Estimates for HIV/AIDS epidemic in China. Chin J AIDS STD. 2012; 18: 1–5

[39] Chen S, Lu X, Bai G, et al. Twenty-seven year surveillance of blood transfusion recipients infected with HIV-1 in Hebei Province, China. PLoS One. 2018;13:e0202265. 30110373 10.1371/journal.pone.0202265 PMC6093692

[40] Chen M, Zhu Q, Xing H, et al. The characteristics of pretreatment HIV-1 drug resistance in western Yunnan, China. Epidemiol Infect. 2020;148:e102. 32381145 10.1017/S095026882000093X PMC7315466

[41] Takebe Y, Liao H, Hase S, et al. Reconstructing the epidemic history of HIV-1 circulating recombinant forms CRF07_BC and CRF08_BC in East Asia: the relevance of genetic diversity and phylodynamics for vaccine strategies. Vaccine. 2010;28(Suppl 2):B39–B44. 20510742 10.1016/j.vaccine.2009.07.101

[42] Zhao J, Chen L, Chaillon A, et al. The dynamics of the HIV epidemic among men who have sex with men (MSM) from 2005 to 2012 in Shenzhen, China. Sci Rep. 2016;6:28703. 27352965 10.1038/srep28703 PMC4926087

[43] Li J, Feng Y, Shen Z, et al. HIV-1 transmissions among recently infected individuals in Southwest China are predominantly derived from circulating local strains. Sci Rep. 2018;8:12831. 30150680 10.1038/s41598-018-29201-3 PMC6110827

[44] Zhao J, Cai W, Zheng C, et al. Origin and outbreak of HIV-1 CRF55_01B among MSM in Shenzhen, China. J Acquir Immune Defic Syndr. 2014;66:e65–e67. 24662297 10.1097/QAI.0000000000000144

[45] Feng Y, Takebe Y, Wei H, et al. Geographic origin and evolutionary history of China’s two predominant HIV-1 circulating recombinant forms, CRF07_BC and CRF08_BC. Sci Rep. 2016;6:19279. 26763952 10.1038/srep19279 PMC4725877

