# Supplementary material for: Identification of two novel HIV-1 circulating recombinant forms of CRF111_01C and CRF116_0108 in southwestern Yunnan, China
Source: Virulence. 2021 Dec 24;13(1):19–29. doi: 10.1080/21505594.2021.2010399 (PMC9794008; doi:10.1080/21505594.2021.2010399)
Supplement: Supplemental Material [file KVIR_A_2010399_SM3883.docx]

Supplementary Materials

**For** Identification of two novel HIV-1 circulating recombinant forms of CRF111_01C and CRF116_0108 in southwestern Yunnan, China

Mei Ye, Xin Chen, Lin Duo, Jin Ma, Le Cao, Chiyu Zhang and Yong-Tang Zheng

**Supplementary Table 1.** The primers used in the amplification of HIV-1 near full-length genomes in this study.

| **Fragment** | **Primer name** | **5' to 3'** | **Position (HXB2)** |
| --- | --- | --- | --- |
| 5’LTR-*pol*  (A1) | AF1 | TACCCACTGTGTTTTGGATGGTGCTT | 114-139 |
|  | AR1 | AGTGTGAGCAGACCTTTTTCTGGCAT | 3610-3635 |
|  | AF2 | GAGAAAACAACTGCCTGTTACACCCC | 187-212 |
|  | AR2 | TGGTCCTGCCCTTGTTTCTGTACTTC | 3531-3556 |
| *gag*-*pol*  (A2) | msf12b | AAATCTCTAGCAGTGGCGCCCGAACAG | 623–649 |
|  | RT3474R | GAATCTCTCTGTTTTCTGCCAGTTC | 3453–3477 |
|  | f2nst | GCGGAGGCTAGAAGGAGAGAGATGG | 769–793 |
|  | proRT | TTTCCCCACTAACTTCTGTATGTCATTGACA | 3308–3338 |
| *pol*-*env*  (B) | BF1 | AAGGAACCTCCATTCCTTTGGATGGG | 3216-3241 |
|  | BR1 | CAGGTGTATTTCTTGTGGGTTGGGGT | 6457-6482 |
|  | BF2 | GAACTGCCAGAAAAAGACAGCTGGAC | 3282-3307 |
|  | BR2 | ACCCAAGGATCAAAGTCCCCCATTTC | 6269-6294 |
| *vpr*-3’LTR  (C) | CF1 | GGGTGTCAACATAGCAGAATAGGCAT | 5782-5807 |
|  | CR1 | TATTGAGGCTTTAAGCAGTGGGTTCC | 9591-9615 |
|  | CF2 | GATCCTAACCTAGAGCCCTGGAATCA | 5843-5868 |
|  | CR2 | TGGTCTAACAAGAGAGACCCAGTACA | 9533-9558 |

**Supplementary Table 2.** The molecular clock signal analysis of each dataset.

| **Dataset** | **Correlation coefficient** | **Residual mean squared** |
| --- | --- | --- |
| CRF111_01C-I-*pol*-_CRF01_AE_ | 0.756 | 1.1548E-4 |
| CRF111_01C-II-*env*-_C_ | 0.5627 | 3.8625E-3 |
| CRF111_01C-III-*env*-_CRF01_AE_ | 0.8217 | 2.562E-4 |
| CRF116_0108-I-*pol*-_CRF08_BC_ | 0.6007 | 9.6173E-5 |
| CRF111_0108-II-*env*-_CRF01_AE_ | 0.7629 | 1.1593E-3 |

**Supplementary Table 3.** The marginal likelihoods of different combinations of clock model and tree prior using path sampling (PS)/stepping-stone sampling (SS) approaches.

| **Dataset** | **Molecular clock model** | **Tree prior** | **PS** | **SS** |
| --- | --- | --- | --- | --- |
| CRF111_01C-I-*pol*-_CRF01_AE_ | Strict clock | Constant size | -14349.62 | -14352.12 |
|  | Strict clock | Exponential growth | -14245.08 | -14248.46 |
|  | Strict clock | Bayesian skyline | -14229.40 | -14236.84 |
|  | Uncorrelated lognormal relaxed clock | Constant size | -14319.95 | -14317.91 |
|  | Uncorrelated lognormal relaxed clock | Exponential growth | -14219.46 | -14223.55 |
|  | **Uncorrelated lognormal relaxed clock** | **Bayesian skyline** | **-14196.75** | **-14202.81** |
| CRF111_01C-II-*env*-_C_ | Strict clock | Constant size | -25316.75 | -25321.51 |
|  | Strict clock | Exponential growth | -25203.22 | -25207.65 |
|  | Strict clock | Bayesian skyline | -25198.10 | -25206.18 |
|  | Uncorrelated lognormal relaxed clock | Constant size | -25279.02 | -25275.76 |
|  | Uncorrelated lognormal relaxed clock | Exponential growth | -25149.45 | -25160.30 |
|  | **Uncorrelated lognormal relaxed clock** | **Bayesian skyline** | **-25130.75** | **-25145.52** |
| CRF111_01C-III-*env*-_CRF01_AE_ | Strict clock | Constant size | -21395.32 | -21401.06 |
|  | Strict clock | Exponential growth | -21265.38 | -21278.98 |
|  | Strict clock | Bayesian skyline | -21251.91 | -21256.55 |
|  | Uncorrelated lognormal relaxed clock | Constant size | -21384.21 | -21380.51 |
|  | Uncorrelated lognormal relaxed clock | Exponential growth | -21255.78 | -21258.98 |
|  | **Uncorrelated lognormal relaxed clock** | **Bayesian skyline** | **-21249.29** | **-21248.11** |
| CRF116_0108-I-*pol*-_CRF08_BC_ | Strict clock | Constant size | -10660.17 | -10659.86 |
|  | Strict clock | Exponential growth | -10593.02 | -10590.99 |
|  | Strict clock | Bayesian skyline | -10528.69 | -10527.23 |
|  | Uncorrelated lognormal relaxed clock | Constant size | -10661.58 | -10657.08 |
|  | **Uncorrelated lognormal relaxed clock** | **Exponential growth** | **-10519.06** | **-10514.14** |
|  | Uncorrelated lognormal relaxed clock | Bayesian skyline | -10520.48 | -10520.62 |
| CRF116_0108-II-*env*-_CRF01_AE_ | Strict clock | Constant size | -27718.96 | -27717.06 |
|  | Strict clock | Exponential growth | -27600.62 | -27605.48 |
|  | Strict clock | Bayesian skyline | -27583.58 | -27592.76 |
|  | Uncorrelated lognormal relaxed clock | Constant size | -27669.18 | -27669.62 |
|  | Uncorrelated lognormal relaxed clock | Exponential growth | -27552.22 | -27550.91 |
|  | **Uncorrelated lognormal relaxed clock** | **Bayesian skyline** | **-27543.10** | **-27546.51** |

The best-fitting tree prior and molecular clock model combinations are indicated in bold font for each dataset.


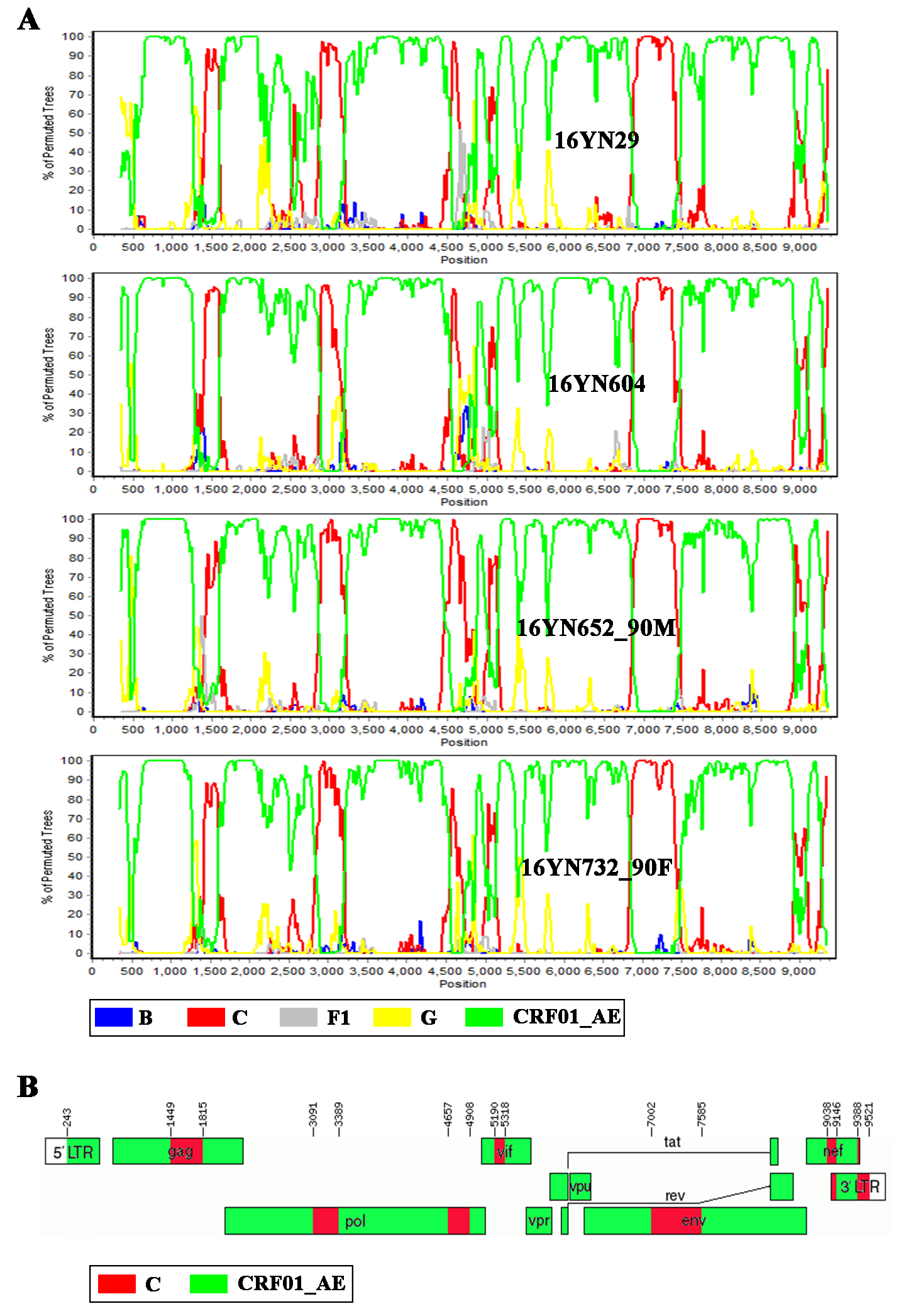


**Supplementary Figure 1.** Bootscan plots (**A**) and genomic structural map (**B**) of CRF111_01C.

**Supplementary Figure 2.** The maximum likelihood trees based on the sub-regions of CRF111_01C.


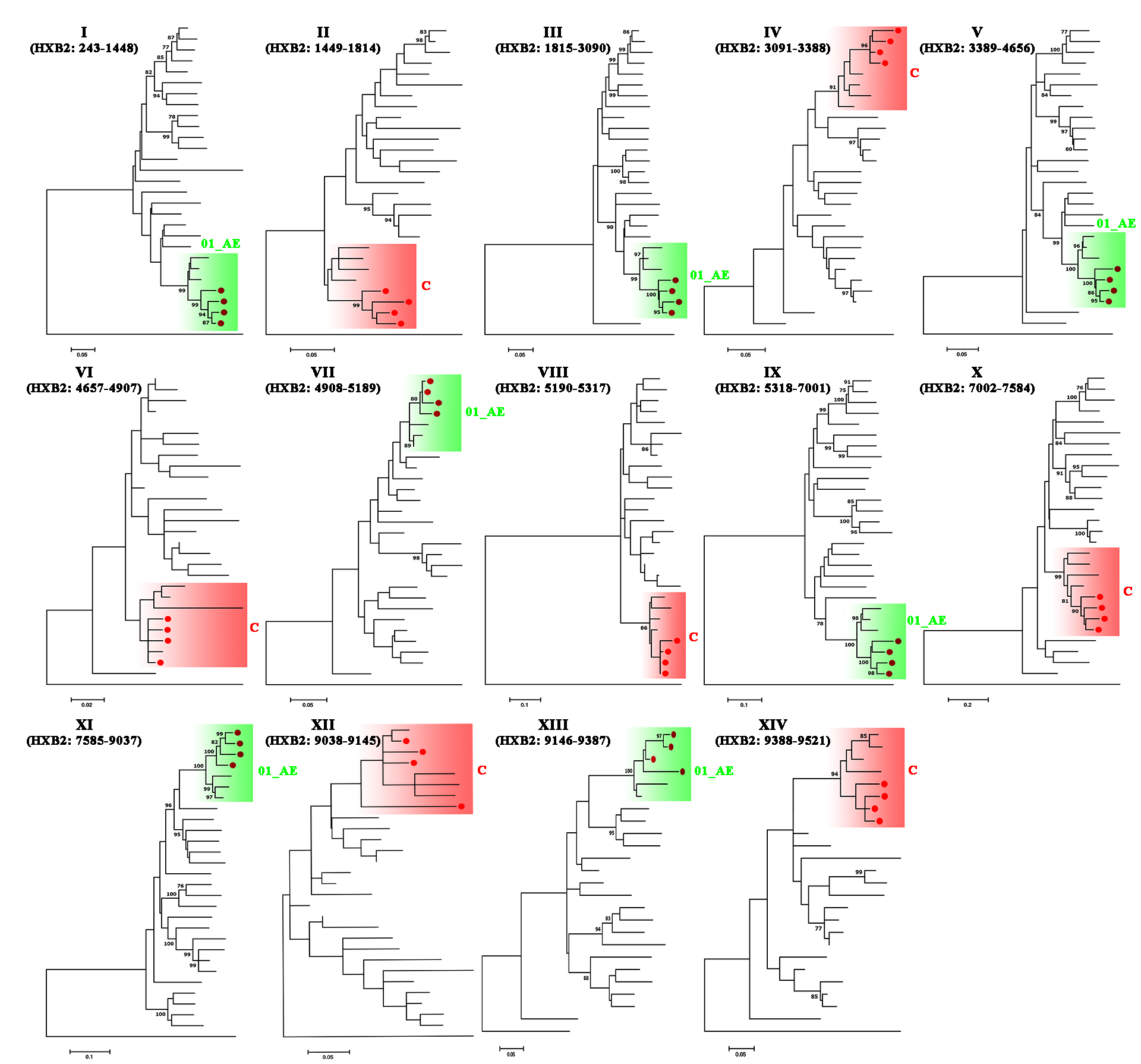

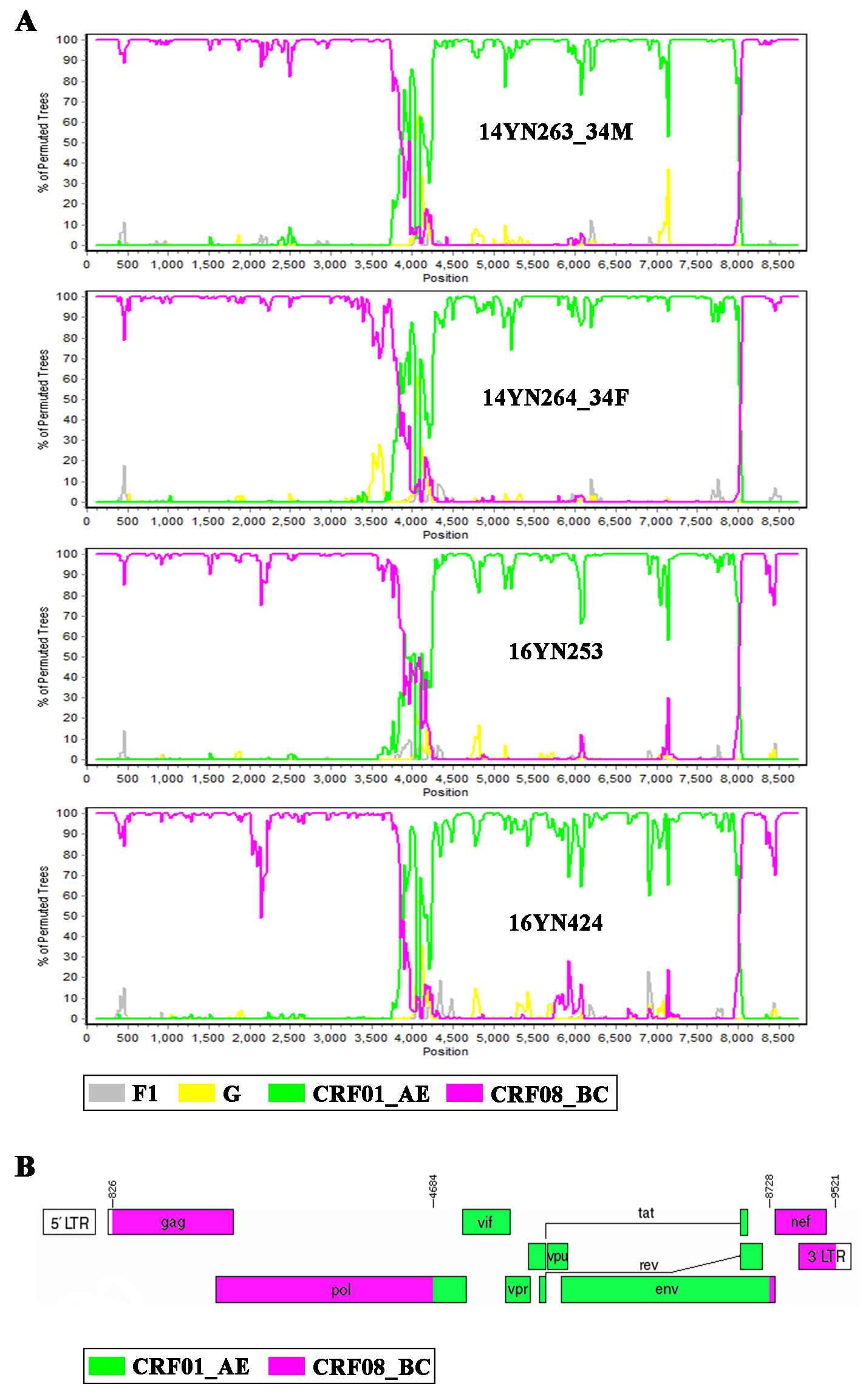


**Supplementary Figure 3.** Bootscan plots (**A**) and genomic structural map (**B**) of CRF116_0108.


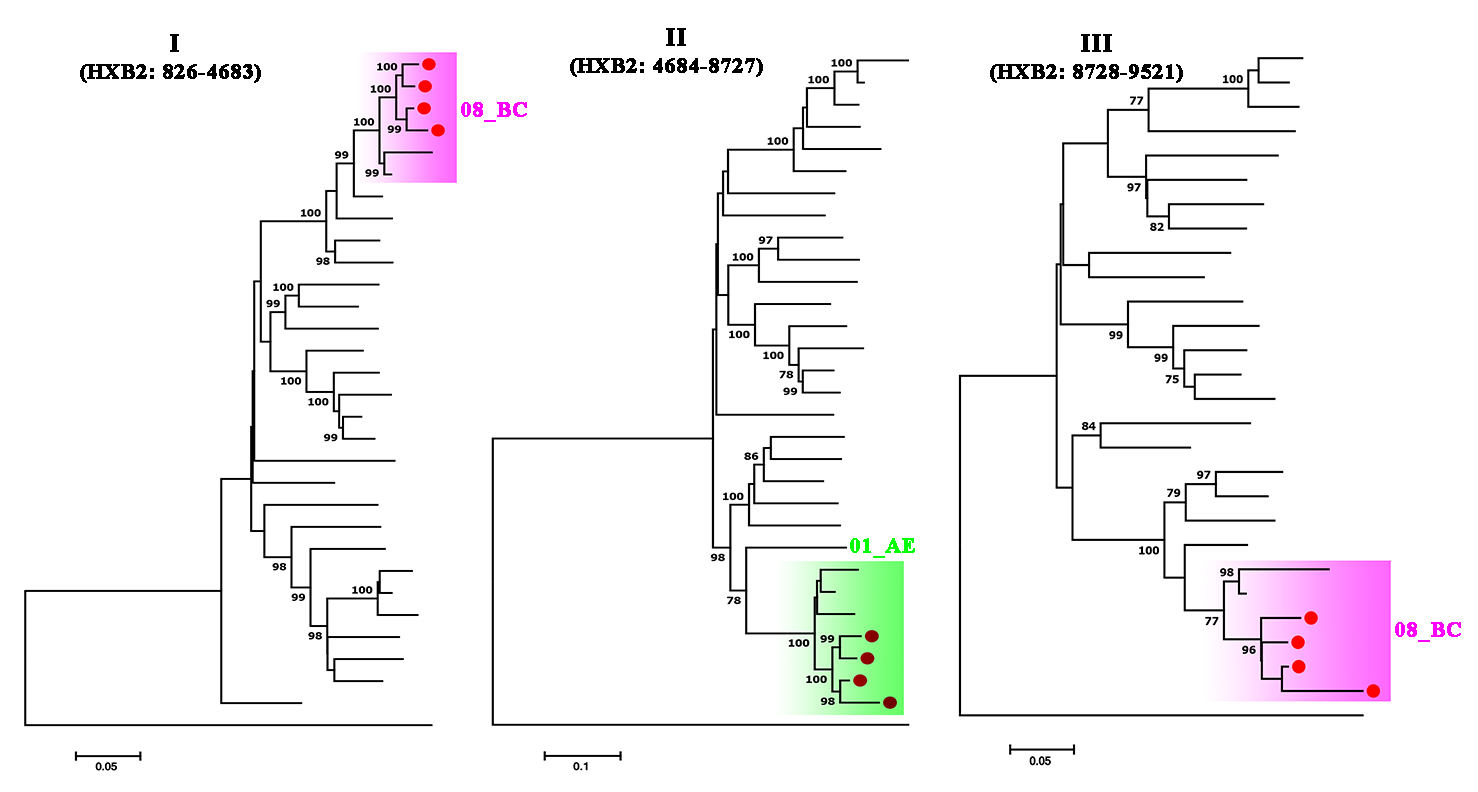


**Supplementary Figure 4.** The maximum likelihood trees based on the sub-regions of CRF116_0108.


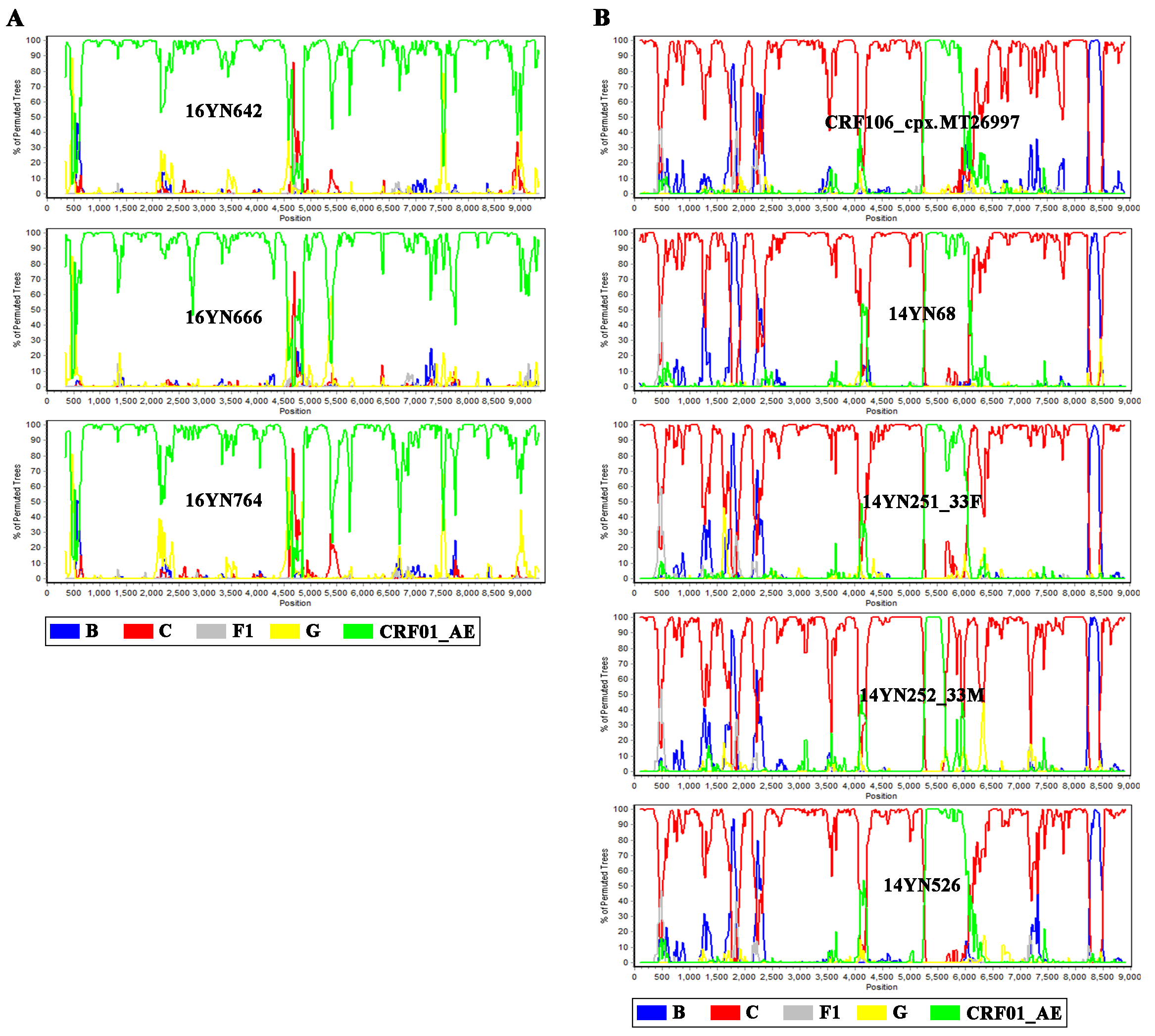


**Supplementary Figure 5.** Bootscan plots of the other 7 HIV-1 near full-length genomic sequences.
